# Gene Expression in Cord Blood and Tuberculosis in Early Childhood: A Nested Case-Control Study in a South African Birth Cohort

**DOI:** 10.1093/cid/ciad268

**Published:** 2023-05-05

**Authors:** Carly A Bobak, Maresa Botha, Lesley Workman, Jane E Hill, Mark P Nicol, John W Holloway, Dan J Stein, Leonardo Martinez, Heather J Zar

**Affiliations:** Department of Biomedical Data Science, Dartmouth College, Hanover, New Hampshire; Department of Paediatrics and Child Health, Red Cross War Memorial Children's Hospital and South African Medical Research Council Unit on Child and Adolescent Health, Cape Town, South Africa; Department of Paediatrics and Child Health, Red Cross War Memorial Children's Hospital and South African Medical Research Council Unit on Child and Adolescent Health, Cape Town, South Africa; School of Biomedical Engineering and the School of Chemical and Biological Engineering, University of British Columbia, Vancouver, Canada; Marshall Centre, Division of Infection and Immunity, School of Biomedical Sciences, University of Western Australia, Perth, Australia; Division of Medical Microbiology, University of Cape Town, South Africa; Human Development and Health, Faculty of Medicine, University of Southampton; National Institute for Health and Care Research Southampton Biomedical Research Center, University Hospital Southampton, United Kingdom; Department of Psychiatry and Mental Health, University of Cape Town; Unit on Risk and Resilience in Mental Disorders, South African Medical Research Council; Neuroscience Institute, University of Cape Town, South Africa; Department of Epidemiology, School of Public Health, Boston University, Massachusetts; Department of Paediatrics and Child Health, Red Cross War Memorial Children's Hospital and South African Medical Research Council Unit on Child and Adolescent Health, Cape Town, South Africa

**Keywords:** tuberculosis, pediatrics, transcriptomics

## Abstract

**Background:**

Transcriptomic profiling of adults with tuberculosis (TB) has become increasingly common, predominantly for diagnostic and risk prediction purposes. However, few studies have evaluated signatures in children, particularly in identifying those at risk for developing TB disease. We investigated the relationship between gene expression obtained from umbilical cord blood and both tuberculin skin test conversion and incident TB disease through the first 5 years of life.

**Methods:**

We conducted a nested case-control study in the Drakenstein Child Health Study, a longitudinal, population-based birth cohort in South Africa. We applied transcriptome-wide screens to umbilical cord blood samples from neonates born to a subset of selected mothers (N = 131). Signatures identifying tuberculin conversion and risk of subsequent TB disease were identified from genome-wide analysis of RNA expression.

**Results:**

Gene expression signatures revealed clear differences predictive of tuberculin conversion (n = 26) and TB disease (n = 10); 114 genes were associated with tuberculin conversion and 30 genes were associated with the progression to TB disease among children with early infection. Coexpression network analysis revealed 6 modules associated with risk of TB infection or disease, including a module associated with neutrophil activation in immune response (*P* < .0001) and defense response to bacterium (*P* < .0001).

**Conclusions:**

These findings suggest multiple detectable differences in gene expression at birth that were associated with risk of TB infection or disease throughout early childhood. Such measures may provide novel insights into TB pathogenesis and susceptibility.

## Teaser

Gene expression in umbilical cord blood demonstrates associations with tuberculosis infection and disease in early life. Differentially expressed genes overlap with known diagnosis and risk signatures. Implicated biological mechanisms include neutrophil activation and defense responses to bacteria.

## Graphical Abstract

**Graphical Abstract:**
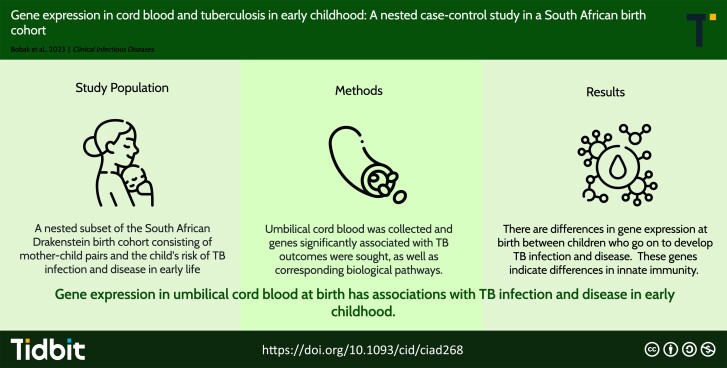
This graphical abstract is also available at Tidbit: https://tidbitapp.io/tidbits/gene-expression-in-cord-blood-and-tuberculosis-in-early-childhood-a-nested-case-control-study-in-a-south-african-birth-cohort

**(See the Editorial Commentary by Suliman et al. on pages 450–2.)**

Approximately 1 million children develop tuberculosis disease (TB) every year, substantially contributing to global pediatric morbidity and mortality [1, 2]. Most children who develop TB are <5 years of age, an age group especially difficult to diagnose [1]. Identifying children who are likely to develop TB based on exposure status and underlying biology is of critical importance to administer targeted preventive therapy and reduce morbidity and mortality [3–5]. RNA transcriptional profiles have been increasingly used for diagnosis and assessment of TB risk among adults and children [6–9]. The relationship between maternal environment in pregnancy and TB risk of offspring is less well understood. Transcriptional analysis of cord blood may potentially provide insight into immune mechanisms that determine risk of TB infection in early childhood.

Whether children differentially express genes compared to adults in relation to TB risk is debated [4]. Gene signatures for diagnosis of pediatric TB have shown specific transcriptomic profiles associated with microbiologically confirmed or clinically diagnosed TB [7]. A small study in India found that children with TB had distinct gene signatures compared to signatures typically used in adults [9]. There are limited data on gene expression profiles predicting TB infection or disease in children, especially from high-TB-burden countries. However, both inherited genetic variation [10] and maternal environmental exposures (both preconceptionally and during pregnancy) are associated with offspring immunity and risk of respiratory infection [11–13]. Identification of gene expression signatures at birth associated with TB risk in childhood may assist our understanding of prenatal factors associated with offspring immunity, allowing for strengthened strategies to prevent TB.

We investigated the relationship between gene expression in cord blood among children who did and did not develop TB infection or disease during early childhood from a prospective birth cohort study in Cape Town, South Africa.

## METHODS

### Participants and Study Design

In a prospective, South African birth cohort, the Drakenstein Child Health Study, we followed children from birth through 5 years of age as described previously [14–17]. In brief, pregnant women between 20 and 28 weeks of gestation were enrolled at community clinics in the Drakenstein area. Exclusion criteria were <18 years of age or intention to leave the area within 1 year. All deliveries occurred at a central hospital, Paarl Hospital, where cord blood was collected by trained staff. Infants were given BCG vaccination at birth (Denmark strain), per national policy. Active surveillance systems for lower respiratory tract illness and TB were established. Children were followed for TB infection and disease until 5 years of age.

Tuberculin skin tests (TSTs) were obtained at 6, 12, 24, 36, 48, and 60 months of age and at the time of lower respiratory tract infection or suspected TB as previously reported [15, 16]. TST conversion was defined as an induration reaction >10 mm in children without human immunodeficiency virus (HIV) or >5 mm in participants with HIV. Repeat testing was not conducted on children with any tuberculin response (ie, >0 mm induration) to minimize potential for tuberculin boosting. Children with positive TST were further screened for TB and referred for preventive therapy.

To diagnose TB, children with a positive TST or who were clinically suspected to have TB were investigated using induced sputum done by trained study staff in duplicate for smear, mycobacterial polymerase chain reaction (Xpert MTB/RIF, Cepheid, Sunnyvale, California), and liquid culture [15, 18]. Chest radiographs were taken in all children suspected of TB and were read and reported by an experienced clinician; TB was diagnosed by experienced healthcare providers in local TB community clinics. We used standardized consensus definitions for diagnostic classification of TB [19]: confirmed TB, unconfirmed TB, and unlikely TB. Diagnoses of TB in this cohort consist of both confirmed and unconfirmed TB.

### Cord Blood Collection, RNA Isolation, and Gene Expression Data Processing

A subset of the cohort was selected for transcriptional profiling using biobanked umbilical cord blood samples as previously described [20]. Samples were collected after delivery by clamping, cutting, and draining umbilical cords into kidney dishes. Blood was collected and stored at −80°C in PAXgene RNA tubes. An IlluminaHT-12 v4 beadchip array was used to obtain row probe intensity values. Samples were previously randomized within batches, based on demographics (ie, sex, maternal diagnoses, maternal alcohol and tobacco use, and mode of delivery) to reduce potential for batch effects [20]. We conducted a case-control study of all previously processed samples nested within the cohort. We defined cases as (1) children who converted their TST over follow-up and (2) children diagnosed with TB. Controls were participants who did not develop TB or convert their TST over follow-up. Children with missing TB outcomes were excluded.

Umbilical cord blood samples, RNA collection, and gene expression array processing were done as previously described [20] and are further detailed in the Supplementary Methods.

Differential gene expression (DGE) analysis was conducted using the *limma* package [21]. DGE was used to identify genes that were significantly differentially expressed for 3 outcomes: (1) infants who did and did not convert their TST before 3 years of age (the former herein referred to as “early converters”); (2) infants who did and did not develop TB before 5 years of age; and (3) among early converters only, infants who did and did not develop TB by 5 years of age. We considered current maternal smoking status adjusted for HIV as a fourth outcome. We employed an exploratory significance threshold of α = .005 for DGE [20, 22] and used Gene Set Enrichment Analysis (GSEA) to map genes to biological pathways [23]. A pathway *z* score was assigned to each sample as in [24]. Weighted gene coexpression network analysis (WGCNA) [25] was conducted to identify and characterize gene modules for their associations with each of the 3 TB outcomes. We used gProfiler [26] to identify enriched pathways within each module, and visualized these using an enrichment map [27]. CIBERSORTx was used to identify absolute abundance of immune cells using transcriptional data [28]. Identified genes, pathways, and modules were compared to 2 cohorts of pediatric TB patients in Kenya and Malawi [7]. Full details on the analytic pipeline are included in the Supplementary Methods.

All computational code is available at https://github.com/CarlyBobak/TBCordBlood. Raw expression data are publicly available through the Gene Expression Omnibus (accession number GSE114852) [29].

## RESULTS

Of 144 biobanked cord blood samples, 131 (91.0%) children had available TST and TB diagnosis data and were included in this analysis. Among these, all children were followed for 5 years with no loss to follow-up or death. Maternal HIV occurred in 25.2%, self-reported smoking in 28.2%, while prior maternal TB diagnosis was reported in 3.8% of all participants. The population was predominantly of low household income. In total, 25.2% were HIV exposed; however, no children had HIV. Median weight-for-age *z* score and height-for-age *z* score at 5 years were −0.07 (interquartile range [IQR], −1.21 to 1.64) and −0.07 (IQR, −1.21 to 1.64), respectively. In total, 10 (7.6%) children received preventive therapy.

Among included children, 26 (19.8%) were early converters while 14 (10.7%) developed TB prior to 5 years of age. Among converters, 10 of 26 (38.5%) subsequently developed TB by 5 years of age.

There were no statistically significant differences between tuberculin converters and nonconverters in relation to maternal HIV status, maternal TB during pregnancy, self-reported history of maternal TB, TB in the household 1 year prior to enrollment, or maternal smoking (Table 1). There were no statistically significant differences between children who did and did not develop TB disease in relation to sex, birthweight, or duration of breastfeeding, while self-reported maternal smoking during pregnancy approached statistical significance, with a higher percentage of smoking mothers represented among children with TB (50% vs 26.6% among TB progressors and nonprogressors; *P* = .07) (Supplementary Table 1).

**Table 1.** Clinical Measures for All Included Study Participants (N = 131) by Tuberculin Skin Test Conversion in Infants

Characteristic	Early TST Converters (n = 26)	Children Who Did Not Convert Their TST (n = 105)	Overall (N = 131)	*P* Value
Maternal characteristics				
Maternal HIV infected (%)	3 (11.5)	30 (28.6)	33 (25.2)	.12
TB treatment during pregnancy (%)	1 (3.8)	4 (3.8)	5 (3.8)	1
Prior maternal TB (%)	0 (0)	5 (4.8)	5 (3.8)	.57
TB in household 1 y prior enrollment (%)	2 (7.7)	14 (13.3)	16 (12.2)	.64
Maternal smoking (%)	9 (34.6)	28 (26.7)	37 (28.2)	.49
Socioeconomic status score, median (IQR)	−0.11 (−1.58 to 1.27)	−0.07 (−1.63 to 1.27)	−0.11 (−1.58 to 1.26)	.456
Household income, rand per month				.579
<1000 (%)	13 (50.0)	41 (39.1)	54 (41.2)	
1000–5000 (%)	10 (38.5)	47 (44.8)	57 (43.5)	
>5000 (%)	3 (11.5)	17 (16.2)	20 (15.3)	
Child characteristics				
Female sex (%)	11 (42.3)	47 (44.8)	58 (44.3)	1.0
Birthweight, kg, mean (sd)	3.07 (0.46)	3.16 (0.53)	3.14 (0.52)	.38
Breastfeeding, mo, median (min, max)	2 (0, 7)	1 (0, 8)	1 (0, 8)	.14
TB diagnosis <5 y old (%)	10 (38.5)	6 (3.8)	16 (12.2)	<.001
WAZ at 5 y of age, median (IQR)	−0.51 (−1.33 to 0.29)	−0.48 (−1.16 to 0.43)	−0.07 (−1.21 to 1.64)	1.0
HAZ at 5 y of age, median (IQR)	−0.67 (−1.24 to −0.15)	−0.58 (−1.27 to 0.10)	−0.07 (−1.21 to 1.64)	.647

Data are presented as No. (%) unless otherwise indicated.

Abbreviations: HIV, human immunodeficiency virus; HAZ, height-for-age *z* score; IQR, interquartile range; sd, standard deviation; TB, tuberculosis; TST, tuberculin skin test; WAZ, weight-for-age *z* score.

### DGE Analysis Reveals Signatures From Umbilical Cord Blood and Infant TB Outcomes

**Figure 1.**
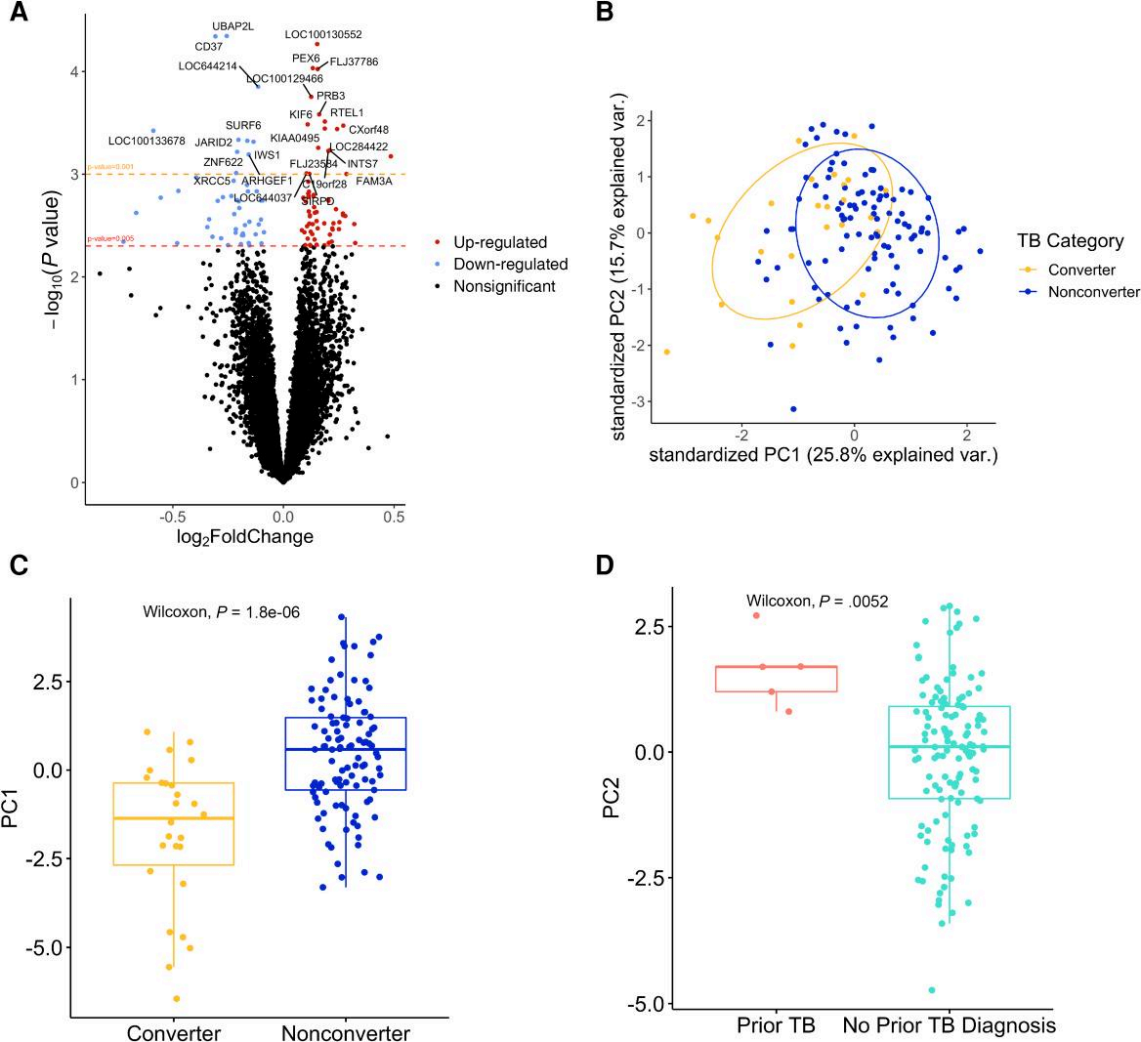
Differential gene expression results between infants with tuberculin skin test (TST) conversion and infants without conversion before 36 mo of age. *A*, Volcano plot showing differentially up- and down-regulated genes between early converters and nonconverting infants. *B*, Principal component analysis using significantly differentially regulated genes from (*A*), where early converters are indicated in yellow and those who did not convert are indicated in blue. *C* and *D*, Boxplots demonstrating statistically significant differences in the first principal component in *B* between infants with and without early TST conversion (*C*) and between mothers with a known prior tuberculosis (TB) diagnosis and mothers with no known prior TB diagnosis (*D*). Abbreviations: PC1, principal component 1; PC2, principal component 2; TB, tuberculosis.

We sought to identify differentially expressed genes (DEGs) in umbilical cord blood between infants who did and did not experience early TST conversion. A total of 114 genes were significant above the exploratory threshold of *P* < .005. Of genes that met the significance threshold, the largest absolute log_2_ fold changes were for *DEFA3* (*P* = .004), *DEFA1* (*P* = .002), *HLA-DQAI* (*P* = .001), and *IFITM3* (*P* = .004; Figure 1*A* ; Supplementary Table 2).

Principal component analysis (PCA) of the significant genes shows a visible trend clustering early converters compared to participants who did not convert (Figure 1*B* and 1*C* ) [30]. The differences in the first principal component (PC1) were statistically significant (*P* = 1.8 × 10^−6^) [31]. PC1 also separated TB progressors from nonprogressors (*P* = .047). The second principal component (PC2) demonstrated statistically significant differences between mothers who had and did not have a prior TB diagnosis before pregnancy (*P* = .0052); this analysis had very small sample size of mothers in the prior TB group (n = 5; Figure 1*D* ).

**Figure 2.**
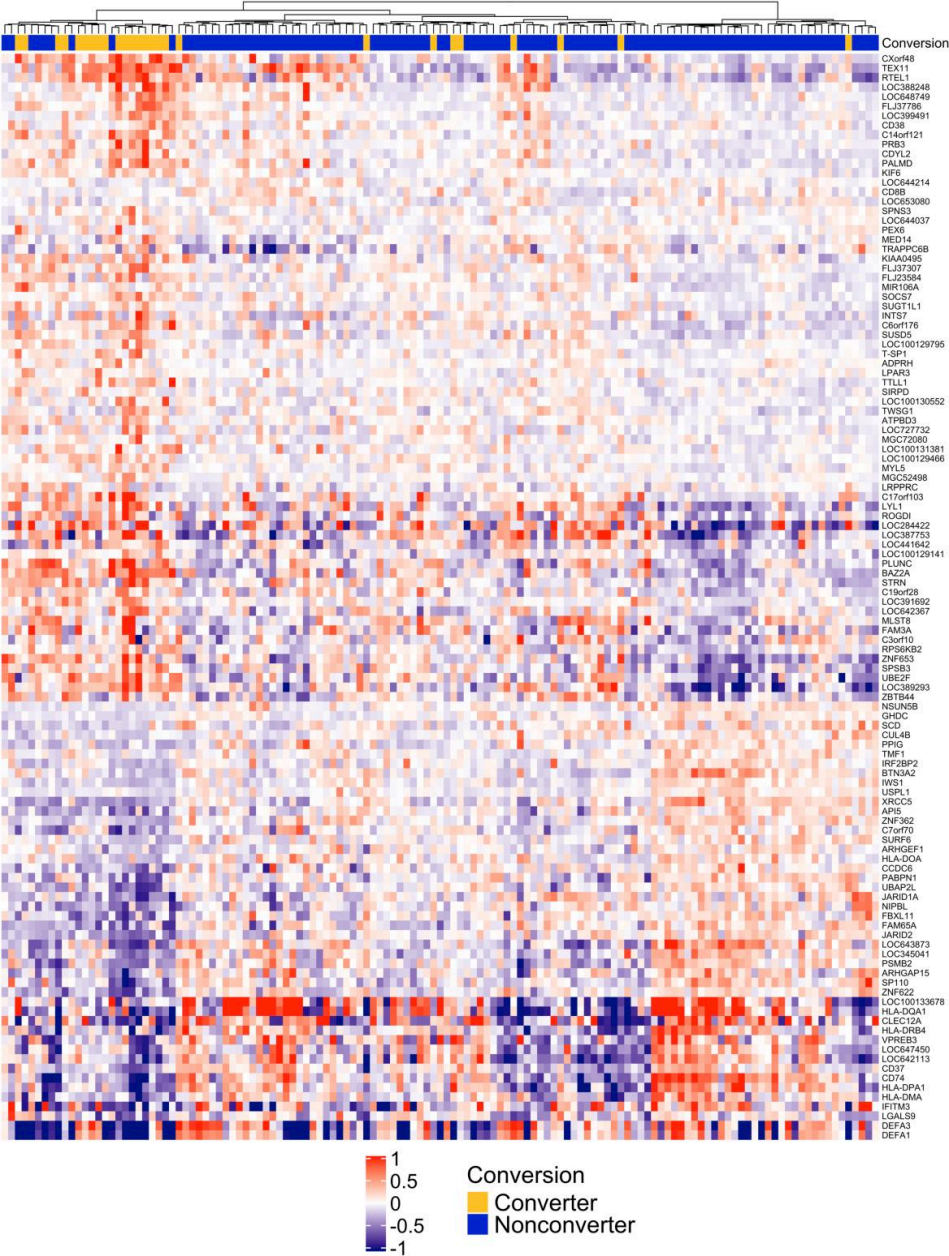
An unsupervised heatmap with infants who did (blue) or did not (yellow) convert their tuberculin skin test prior to 36 mo of age. Each row represents a significantly associated gene (*P* < .005) and each column is 1 umbilical cord blood sample. Expression values were median centered. Both columns and rows were clustered using Canberra distance.

Median centered expression values are displayed using a heatmap with unsupervised hierarchical clustering in Figure 2 [32]. Clustering among early converters was present using this umbilical cord gene expression signature. This finding suggests there are distinct gene expression differences associated with greater susceptibility to TB infection.

When focusing our analysis on incident TB, we identified 60 genes that were statistically significant (*P* < .005) between TB progressors and nonprogressors. The most significant genes included *SULT1A3* (*P* = 8.05 × 10^−5^), *HMBS* (*P* = 1.50 × 10^−4^), and *NCOA3* (*P* = .002) (a full list of these results is shown in Supplementary Table 3 and Supplementary Figure 1).

**Figure 3.**
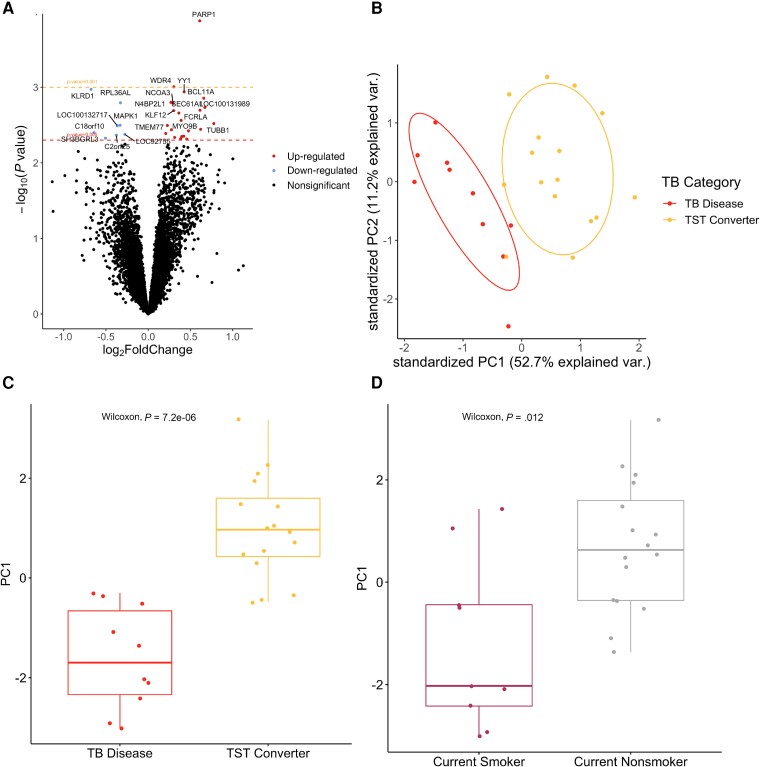
Differential gene expression results between infants who were diagnosed with tuberculosis (TB) before the age of 5 from those without a TB diagnosis among those who experienced tuberculin skin test conversion before 36 mo of age. *A*, Volcano plot showing the effect size by the -log_10_*P* value. *B*, Principal component analysis using just the significantly differentially expressed genes. *C*, Boxplot showing statistically significant differences along the first principal component between children diagnosed with TB before age 5 and those who were not. *D*, Boxplot showing statistically significant differences between mothers who were smokers at the time of enrollment and mothers who were not smokers at time of enrollment. Abbreviations: PC1, principal component 1; PC2, principal component 2; TB, tuberculosis; TST, tuberculin skin test.

In an analysis of TB disease in the first 5 years restricted to early TST converters, we found 30 associated genes, where the most significant included *PARP1* (*P* = 9.84 × 10^−5^), *WDR4* (*P* = 5.34 × 10^–4^), and *KLRD1* (*P* = .002) (Figure 3*A* ; Supplementary Table 4). In PCA (Figure 3*B* ), there were clear differences along PC1 (Figure 3*C* ), and these differences were statistically significant (*P* = 7.2 × 10^−6^). PC1 was also associated with maternal smoking status (*P* = .012). In an unsupervised hierarchical clustering analysis, we observed a strong clustering of 6 infants, where 5 of 6 of those infants had mothers who were current smokers at enrollment (Supplementary Figure 2).

**Figure 4.**
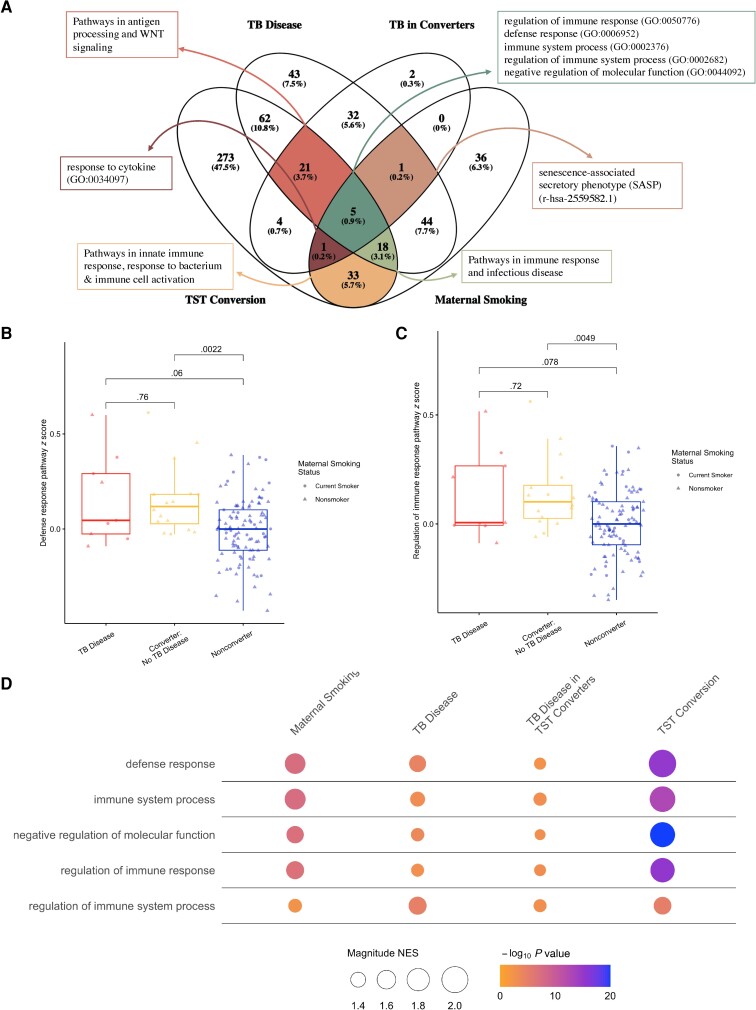
Results from a pathway overlap analysis of all 3 tuberculosis (TB) hypotheses and human immunodeficiency virus–adjusted maternal smoking. Pathways were identified using Gene Set Enrichment Analysis [23]. *A*, Venn diagram of all pathways that overlapped between each differential gene expression (DGE) analysis. *B*, Pathway response *z* score of the defense response pathway (GO:0006952) across TB outcomes. *C*, Pathway response *z* score of the regulation of immune response pathway (GO:0002682) across TB outcomes. *D*, Bubble plot illustrating normalized effect size and *P* values of all pathways that were either enriched or depleted in each DGE analysis. Abbreviations: GO, Gene Ontology; NES, normalized effect size; TB, tuberculosis; TST, tuberculin skin text.

A DGE and subsequent GSEA revealed that pathways related to immune response, response to bacterium, and immune cell activation were overlapping across all TB outcomes and maternal current smoking status (Figure 4*A* and 4*D* ). These pathways significantly differentiate between TB outcomes (Figure 4*B* and 4*C* ). Further smoking DGE and GSEA results and an enrichment map are available in Supplementary Tables 5–9 and Supplementary Figure 3.

### Biologically Relevant Modules Reveal Meaningful Networks of Gene Expression for TST Conversion and Diagnosis of TB in Young Children

**Figure 5.**
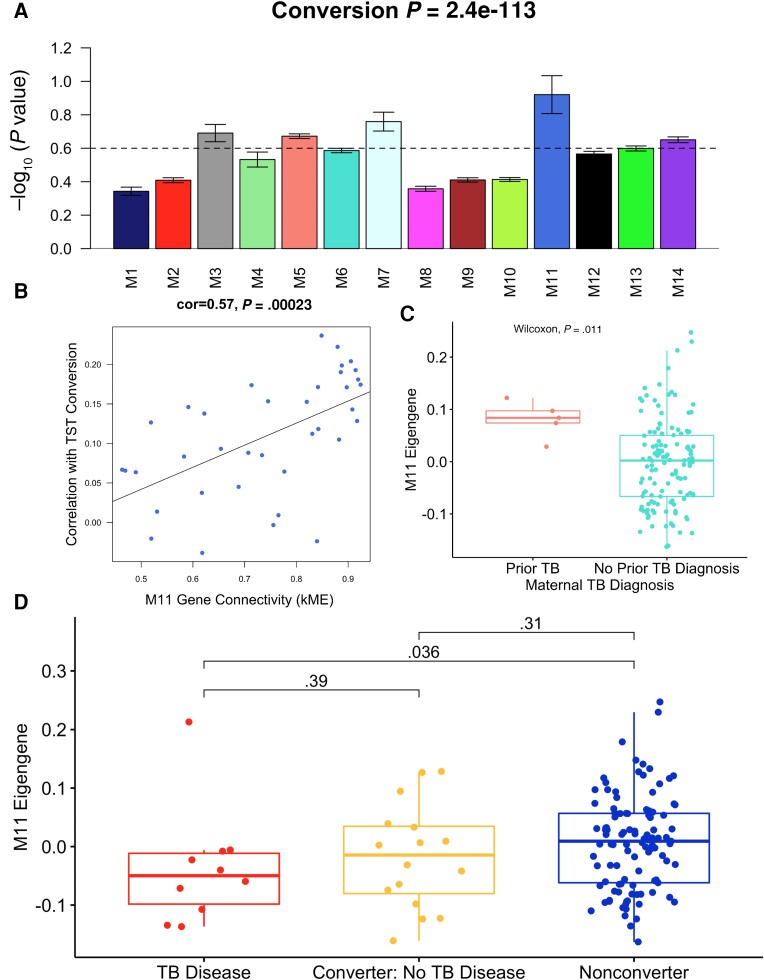
Weighted gene coexpression network analysis. *A*, The 14 discovered gene expression modules and their association with early tuberculin skin test (TST) conversion. Error bars represent 1 standard error on either side of the mean. *B*, Regression between the significance of genes in the M11 module with early TST conversion and the gene connectivity within the module. *C*, Boxplot of the M11 module eigengene by maternal prior tuberculosis (TB) diagnosis. *D*, Boxplot of the M11 eigengene between early TST converters, early converters who developed TB, and children who did not convert their TST in early childhood and who did not develop TB within their first 5 y of age.

We used WGCNA to identify interpretable, biologically relevant co-regulated gene modules. A total of 14 modules were identified (Supplementary Tables 10 and 11), and we evaluated module significance by testing for overrepresentation of disease-related differential gene signatures across all TB-related outcomes and modules. We found that modules M3, M5, M7, M11, and M14 were significantly associated with the early conversion gene expression signature, with M11 having the most significant association (Figure 5*A* ). No modules were related to the development of TB, but M9 was associated with development of TB among children with early conversion (Supplementary Figure 4).

We found a strong correlation between TST conversion and M11 gene connectivity (kME) (Figure 5*B* ). The greater association between gene expression value and early TST conversion, the greater the connection was within the M11 module (correlation = 0.57, *P* = .00023). The M11 eigengene was tested for associations among maternal and child health characteristics. Significant associations were found across several characteristics including maternal prior TB diagnosis (*P* = .01), infant birth weight (*P* = .044), and early TST conversion (*P* = .047) (Figure 5*C* ; Supplementary Figures 5 and 6). Children who developed TB among early converters compared to those who neither converted nor developed disease drove this association (*P* = .036; Figure 5*D* ).

**Figure 6.**
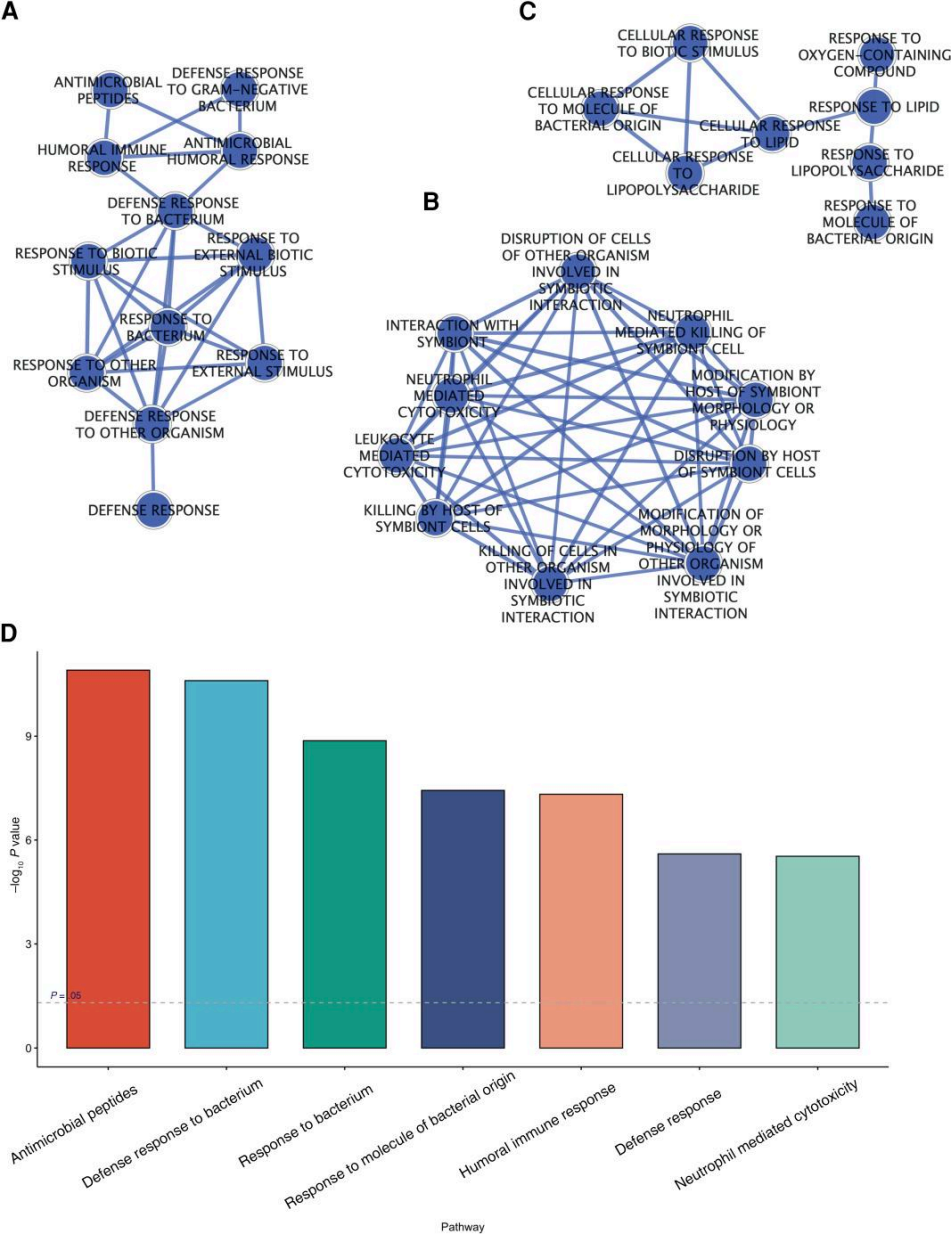
Three extracted subnetworks from the weighted gene coexpression network analysis enrichment results. Nodes represent the biological function, where edges indicate significant overlap of genes associated with the biological functions. Pathways listed are from Gene Ontology, Reactome, Wikipathways, and Kyoto Encyclopedia of Genes and Genomes. *A*, Subnetwork of biological pathways related to the defense response to bacteria. *B*, Subnetwork of responses related to the interaction of the host and symbiont cells. *C*, Subnetwork of cellular responses to molecules of bacterial origins. *D*, Barplot of select pathways plotted against their gProfiler *P* value [26].

We identified functionally enriched biologically interpretable pathways for each module (Supplementary Table 12). The top pathways functionally enriched in the M11 module included neutrophil degranulation, neutrophil activation involved in immune response, and neutrophil-mediated immunity (all from Gene Ontology: Biological Processes [GO:BP]; adjusted *P* values of 1.13 × 10^−22^, 1.29 × 10^−22^, and 2.07 × 10^−22^, respectively). Other pathways include the innate immune system (Reactome; 3.6 × 10^−14^), antimicrobial humoral response, and killing cells of other organisms (GO:BP; *P =* 7.47 × 10^−13^ and *P =* 3.05 × 10^−12^). Select pathways are visualized in Figure 6*D* .

In a full enrichment map, we found overlapping pathways (M5, M9, M11, and M14) across all modules significantly associated with early childhood TST conversion or TB diagnosis (Supplementary Figure 7). We also found 3 extracted subnetworks from the enrichment map using pathways functionally enriched with M11 (Figure 6). These highly linked subnetworks demonstrate major trends in the biological functionality associated with M11 with Figure 6*A* representative of the defensive immune response to bacteria, Figure 6*B* associated with the host interaction with symbiont cells, and Figure 6*C* focused on the cellular response to molecules of bacterial origin.

Using CIBERSORTx cell-type abundance estimation, we found cell-type differences between (1) TB progressors and early TST converters as well as (2) TB progressors and children who did not TST convert. These differences were present in γδ T-cells, which were decreased in TB progressers (*P* = .0016 and *P* = .0114) and mast cells (also decreased; *P* = .0292 and *P* = .0006). Neutrophils were decreased in TB progressors compared to children who did not TST convert (*P* = .045); this was not statistically significant when comparing TB progressors and children who TST converted (*P* = .1073). B cells were decreased in TST converters compared to nonconverters (*P* = .0396). Full CIBERSORTx results are available in Supplementary Table 13.

### DEGs and Modules in Cord Blood Are Present at the Time of Diagnosis

Across 2 independent pediatric TB cohorts that measured gene expression in whole blood at time of diagnosis [7], we found that 78 of 128 (60.9%) measured cord blood genes were differentially expressed (adjusted *P* < .05). Similarly, the M11 module was both preserved and high quality in both the Kenyan and Malawi cohorts (preservation *P* = 1.37 × 10^−28^ and *P* = 7 × 10^−20^; quality *P* = 3.07 × 10^−40^ and *P* = 3.01 × 10^−53^). Additional validation can be found in the Supplementary Results and Supplementary Figure 8.

## DISCUSSION

In this South African birth cohort study following infants through 5 years old in an area of high TB prevalence, we found several novel gene expression profiles from umbilical cord blood that differentiated children at risk of TST conversion and incident TB. Several identified genes have established associations in TB pathogenesis, predominantly in adults [7, 8, 33–41]. These studies have traditionally assumed gene expression changes due to TB exposure, infection, or disease. In this work, we show that differences in gene expression disease may occur prior to birth, suggesting the possibility of genetic or epigenetic predisposition or possible in utero exposure.

The most important DEGs in each of our 3 signatures have been previously associated with TB disease in children [7, 8] as well as other TB immune responses in adults and cell culture and murine models [34–40]. Genes that best predicted TST conversion included *DEFA1* and *DEFA3*, both proposed biomarkers for detecting TB from latent TB in children [7, 8]; *HLA-DQAI,* which was associated with protection against pulmonary TB in a prior meta-analysis of TB infection in adults [34]; and *IFITM3*, which is implicated in the restriction of mycobacterial growth [35]. Our top DEGs for TB progression include *SULT1A3*, which is associated with treatment response for TB in adults [36], and *NCOA3*, which was differentially expressed in microRNA in adults with TB compared with adults hospitalized without a TB diagnosis [37]. Among early TST converters, the top DEGs between those who did and did not progress to TB include *PARP1* and *KLRD1*. *PARP1* has been implicated in mouse experiments, plays a fundamental role in the host response to TB, and is hypothesized to contribute to the sex differences in response to TB [38]. *KLRD1* has been demonstrated to be a potential T-cell–linked biomarker in the progression to TB in mice and macaques [39, 40]. Furthermore, it has previously been associated with natural killer cell function in both influenza and TB [33, 41].

Of note, both *PARP1* and *KLDR1* have known associations with exposure to cigarette smoke, with PARP1 being implicated in both cellular senescence and lung DNA damage [42, 43]. Active and passive smoking have long been associated with TB [44, 45], and our previous work with this cohort found an association between maternal smoking and subsequent TB risk [16]. This is the first study to show that umbilical cord gene expression changes are associated both with maternal smoking status and development of TB among children with early TST conversion. The overlap in genes linked to smoking and TB, due to our temporal sampling method for exposure and diagnosis, reflects a biological mechanism that partly clarifies the link between maternal smoking and childhood TB outcomes. While an association between maternal smoking and TB progression did not reach statistical significance (*P* = .07) given the small sample size of this study, the observed differences may provide insight on the factors that increase TB risk in children. Further studies are needed to characterize this relationship.

Sampling gene expression at birth provides a unique opportunity to study TB pathogenesis preceding exposure. We found a collection of gene signature modules which are associated with TB infection and disease. The most significant of these modules was M11, where pathway analysis indicated genes that primarily implicate neutrophil activation. Previous diagnostic signatures have highlighted neutrophil-driven transcriptional changes as critical in adults with TB [46]. Neutrophils are a critical part of innate immunity and are the primary attackers of bacterial infections, and thus may be important for protection against TB [47]. High neutrophil counts in peripheral blood were highly protective of TB among household contacts [48]. Given that the M11 gene module was negatively associated with TST conversion and TB within early converters, this adds further evidence that neutrophil activation is important in TB protection. This result was further supported using cell-type abundance estimates from CIBERSORTx, indicating that circulating neutrophils were lower among TB progressors compared to children who never TST converted or developed TB.

Clusters of pathways implicated by the M11 gene module include those that are representative of the defensive immune response to bacteria and cellular response to molecules of bacterial origin. Similar pathways are often observed in gene expression studies in patients with TB [49], suggesting that changes in these pathways are already present at birth and may influence TB infection and disease in early childhood.

Limitations of this study include the small sample size. The DGE results specifically are underpowered and should be interpreted cautiously. Modular support with WGCNA provides additional evidence that meaningful and interpretable biology is occurring during or prior to birth that influences early childhood TB outcomes. Future large-scale work with additional clinical and biological data from both mother and infant is essential for further elucidating thesemechanisms, particularly in addressing possible confounding from unmeasured characteristics. Additionally, *Mycobacterium tuberculosis* infection is likely a heterogenous state and it is possible some of our converters may have been false positives or in early stages of disease. BCG boosting might lead to false-positive conversion results; to address this issue, we used a conservative conversion cutoff. Furthermore, any child with a positive skin test reaction did not have a repeat skin test [50]. Additionally, CIBERSORTx estimates of cell-type abundances have not been validated on umbilical cord data and should be interpreted carefully. We are also unable to distinguish whether cord blood gene expression is on the causal pathway to postnatal TB infection or is a biomarker of other exposures that directly alter offspring infection risk (eg, maternal smoking [11], HIV [12], and stress [13]). However, these are mitigated by key strengths, which include intensive participant follow-up and surveillance for TB infection and disease, as well as excellent phenotyping and cord blood RNA expression measurements. The cohort is representative of many populations with low-income economies, where TB continues to be a major cause of child illness and death.

## Supplementary Data

Supplementary materials are available at *Clinical Infectious Diseases* online. Consisting of data provided by the authors to benefit the reader, the posted materials are not copyedited and are the sole responsibility of the authors, so questions or comments should be addressed to the corresponding author.

## Supplementary Material

ciad268_Supplementary_Data

## Notes

***Acknowledgments.*** We thank the study staff; the clinical and administrative staff of the Western Cape Government Health Department at Paarl Hospital and at the clinics for support of the study; members of the study International Advisory Board for their advice; our collaborators; and the families and children who participated in the study.

***Author Contributions.*** C. A. B. analyzed the data. C. A. B. and L. M. wrote the first draft of the manuscript. H. J. Z. was the principal investigator, obtained funding, conceived and designed the study, and assisted with drafting of the manuscript. M. N. was the lead microbiologist. M. B. provided operational oversight. L. W. provided data support. D. J. S. obtained funding and oversaw RNA expression aspects. All authors contributed to the interpretation of results. All authors reviewed, contributed to, and approved the final manuscript.

***Financial support.*** This work was funded by the Bill & Melinda Gates Foundation (grant number OPP 1017641); South African Medical Research Council (MRC); the National Research Foundation (South Africa); and the Wellcome Trust. C. A. B. was supported by the Burroughs Wellcome Fund institutional program grant unifying population- and laboratory-based sciences to Dartmouth College (grant number 1014106). J. E. H. received support from Dartmouth College and reports grants from the US National Institutes of Health (NIH), the US Cystic Fibrosis Foundation, the Australian Research Council, and the National Sciences and Engineering Research Council of Canada during the completion of this study. H. J. Z. reports grants from the Bill & Melinda Gates Foundation, the NIH, the Wellcome Trust UK, and the MRC South Africa, National Research Foundation (South Africa), during completion of the study. D. J. S. reports support from NIH and MRC South Africa. L. W. received support from the Bill & Melinda Gates Foundation (grant number OPP 1017641) and the MRC South Africa, National Research Foundation South Africa. M. N. was supported by the Bill & Melinda Gates Foundation and the Australian National Health and Medical Research Council (grant number 1174455; payments made to institution). L.M. was funded by the National Institutes of Health (grant number 5K01AI156022).
