# Supplementary material for: Gene Expression in Cord Blood and Tuberculosis in Early Childhood: A Nested Case-Control Study in a South African Birth Cohort
Source: Clin Infect Dis. 2023 May 5;77(3):438–49. doi: 10.1093/cid/ciad268 (PMC10425199; doi:10.1093/cid/ciad268)
Supplement: ciad268_Supplementary_Data [file ciad268_supplementary_data.zip › Supplemental Methods.docx]

Gene expression in cord blood and tuberculosis in early childhood:

A nested case-control study in a South African birth cohort

Carly A. Bobak, Maresa Botha, Lesley Workman, Jane E. Hill, Mark Nicol, John W. Holloway, Dan J Stein, Leonardo Martinez, Heather J Zar

**Supplemental Methods**

*Cord blood collection, RNA isolation and microarray hybridization*

RNA was collected and processed from UCB as described previously[1]. Samples were collected post infant delivery but prior to placental delivery. Umbilical cords were clamped and cut, then released and drained into kidney collection dishes. Blood was collected from the dish using a syringe and stored at -80°C in PAXgene RNA tubes. Staff were specifically trained to collect only UCB and no other external blood. All samples had a Bioanalyzer RNA integrity number (RIN) 7. Complimentary DNA was derived, hybridized, and row probe intensity values were obtained using an IlluminaHT-12 v4 beadchip array.

*Gene expression data processing, quality control and differential expression*

RNA expression data was processed, normalized, and quality treated in a previous study[1]. In short, all samples were background corrected, quantile normalized, and log_2_ transformed using the *limma* package in the R statistical language[1–3]. Outliers were detected and removed using unsupervised hierarchical clustering and principal component analysis.

All novel analyses for this study were conducted in R 3.6.1 (R Core Team, Vienna, Austria)[3].

We used the using the *limma* pakage[2] to conduct differential gene expression (DGE) analysis to identify genes associated with three TB outcomes: (i) infants who did converted their tuberculin skin test (TST) before three years old from those that did not; (ii) infants who developed TB before five years old and those who did not; and (iii) among infants who converted before age 3, those developed TB disease before age 5 from those who did not. We used a significance threshold of  α=0.005 for differential gene expression, reflecting the exploratory nature of this pilot study [1,4].

We used Gene Set Enrichment Analysis (GSEA) [5] to identify pathways which were significantly enriched or depleted among our gene signatures. The log_2_ fold change (FC) and p-values from the DGE analysis were used to rank genes according to

$Rank= sign\left( \log_{2} FC \right)\times\left( -1 \right)\log_{10} \left( p \right)$ (1)

We downloaded the Gene Ontology (GO) process collection of gene sets from the Molecular Signature Database (MSigDB). The R package ‘fgsea’ was used to conduct GSEA, were we set eps=1e-100, minSize=50, and maxSize=1000 [6]. The output after running GSEA was used to create an EnrichmentMap in Cytoscape v.3.9.1 [7]. Overlapping pathways were visualized using <https://bioinfogp.cnb.csic.es/tools/venny/> . The Z-scores at the pathway level were determined by computing the difference between the mean values of upregulated and downregulated genes within each pathway and dataset, as recommended in [8,9]. We adjusted the pathway scores based on the median value observed in the non-asymptomatic control. To assess whether there was a significant difference in these pathway scores between outcomes we employed a Wilcoxon rank sum test [8].

CIBERSORTx was used to identify absolute abundance of immune cells using transcriptional data using the canonical LM22 set and 1000 permutations for significance analysis [9]. Cell fraction categories were then combined to B cells, CD8 T cells, CD4 T cells, gamma delta T cells, NK cells, monocytes, M0 macrophages, dendritic cells, mast cells, and neutrophils. Each class of cells was tested for significantly different proportions between infants with and without early TST conversion, infants with and without a TB diagnoses, and within infants with early TST conversion, infants who activated to active TB status using a two-sided Student’s T-test[10].

*Weighted gene co-expression network analysis*

Weighted gene co-expression network analysis (WGCNA) was conducted as described previously[1,11]. In short, a signed co-expression consensus network using all 10,705 genes and 144 samples was built to detect biologically meaningful gene modules. Absolute Pearson correlation coefficients were calculated for all gene-gene pairs, and values were transformed with exponential weight (β=8) so that the correlation matrix followed a scale-free topology (R^2^=0.9). To detect the biologically meaningful network modules the WGCNA dynamic cut-tree algorithm was used (minimum module size=30, cut-tree height=0.99, deep-split=2, merge module height=0.25). Identified network modules were tested for association with both maternal and infant characteristics including TB related diagnoses. The module eigengene (ME; equivalent to the first principal component of each network module) was used as a representation of the modules for the testing of statistical associations. As well, we used the gene significance (GS; log_10_ p-value from the DGE analysis described above) for each gene in each module to add additional evidence for the maternal and infant outcomes and characteristic tests. Module significance (MS) is calculated as the mean GS within each identified module. We used Wilcoxon rank sum tests to test the differential analysis of ME [8]. We identified hub genes for each module based on intramodular membership (kME) defined as the correlation between gene expression values and ME expression. In each module we define hub genes as those with the highest kME.

*Enrichment analysis*

Functional pathway enrichment was assessed for all biologically relevant modules using gProfiler[12] which uses statistical enrichment analysis to add biological interpretation to gene lists. In this analysis, we considered flat (unordered) gene lists. Our gene lists cross referenced  pathways from KEGG[13,14], REACTOME[15,16], Gene Ontology (GO)[17], WikiPathways[18–20],  TRANSFAC[21], miRTarBase[22,23], Human Protein Atlas (HPA)[24–27], Comprehensive resource of mammalian protein complexes  (CORUM)[28], and the Human phenotype ontology (HP)[29]. P-values were calculated using a cumulative hypergeometric test [30] with g:Profiler’s specific multiple adjustment correction, set counts and sizes (g:SCS)[12]. Any pathway with an adjusted p-value less than 0.05 was considered functionally enriched.

Enriched pathways in modules that were statistically significant in infants were visualized using Enrichment Map [7] in Cytoscape [31]. Nodes represent pathways with enrichment in module genes (FDR q-value<0.1), where edges indicate overlapping genes between modules (similarity cutoff=0.9, similarity is the average of the Jaccard coefficient and overlap coefficient)[7], revealing various subnetworks within each module. Node colors are defined to match the biologically relevant gene module.

*Validation in publicly available pediatric TB studies*

We downloaded two transcriptomic datasets (GSE39939 and GSE39940) which included pediatric TB samples from the Gene Expression Omnibus for validation purposes [32,33]. These datasets include samples collected from whole blood with patients in Kenya and Malawi. We reduced both validation expression matrices to include only those genes uncovered in our cord blood signatures or the M11 gene module. We conducted DGE analysis using ‘limma’ according to two way comparisons of outcomes in the Anderson datasets (TB and LTBI, Other Diseases and LTBI, TB and Other Diseases) [2,32]. We considered genes to be ‘validated’ if there were differentially expressed at an Benjamini-Hochberg adjusted p-value <0.05 in either Anderson cohort, regardless of direction of log_2_ Fold Change given the lack of healthy controls impacting the choice of control group [32,34].

We examined the module preservation of the M11 module in both Anderson datasets using the ‘WGCNA’ package [11]. Summary preservation statistics and summary quality statistics were calculated, and modules were considered high quality and preserved if statistics were $\geq$ 10 and p-values were $\leq$ 0.05. We used the gene connectively originally calculated in the cord blood expression data, and calculated the Pearson correlation with the correlation of genes to the disease outcomes present in Anderson [35]. We recalculated the eigengene within each cohort using the ‘WGCNA’ package, and used a Wilcoxon test to evaluate two way comparisons across disease diagnosis in the Anderson cohorts [8,11,32].

**Supplemental Validation Results**

Across our 3 TB outcomes, 193 unique genes were differentially expressed. 128 of those genes were measured in the Anderson cohorts. In the DGE analysis comparing LTBI to Other Diseases, a total of 42 out of 71 (58.3%) measured genes from out early conversion DGE were significantly expressed between the Kenya and Malawi cohorts. In the DGE analysis comparing TB to LTBI, 23 out of 45 (51%) measured genes from the early TB diagnosis compared to children without a TB diagnosis were differentially expressed between both cohorts. If we consider those children who were diagnosed with TB disease among early TST converters, 10 out of 23 measured genes (43.5%) were differentially expressed. Across both validation sets, and each two way comparison of disease outcomes, a total of 78 out of 128 (60.94%) of genes were differentially expressed.

We investigated the preservation and quality of the M11 module in both Anderson datasets. We found the module to be preserved, with summary preservation Z-scores of : 13.03 (p=1.37x10^-28^) and11.11 (p=7x10^-20^) between the Kenya and Malawi cohort respectively. The module was also of high quality, with summary quality Z-scores of: 20.22 (p=3.07x10-^40^), 23.31(p=3.01x10^-53^). Module gene connectivity as calculated in the cord blood gene expression showed a tendency towards correlation with the correlation of genes and disease outcomes, (cor=0.34, p=0.052 in Kenya; cor=0.39, p=0.027 in Malawi). The module eigengene, recalculated on the 32 module genes measured in Anderson out of the 37 identified in the cord blood, demonstrated an association with the Anderson disease diagnoses (Wilcoxon p-values; LTBI vs TB p=6.6x10^-5^, LTBI vs other disease p=4.4x10^-6^ in Kenya; LTBI vs TB p=5.1x10^-13^, LTBI vs other disease p=6.0x10^-8^, TB vs other disease p=0.0557 in Malawi).

Of note, our analysis measured gene expression in umbilical cord blood at time of birth and Anderson et al. measured gene expression in whole blood at time of diagnosis. Validation of our findings in Anderson et al. is hence difficult as both the exposure to infection and tissue is different. As well, Anderson et al. compared their TB outcomes (LTBI and TB disease) to other infectious diseases while we compared to children who did not convert and/or did not develop TB disease by the age of 5. Thus we anticipated limited agreement between these two datasets and recommend caution when interpreting the results.

**Supplemental Methods References**

1. Breen MS, Wingo AP, Koen N, et al. Gene expression in cord blood links genetic risk for neurodevelopmental disorders with maternal psychological distress and adverse childhood outcomes. Brain Behav Immun [Internet]. Elsevier; **2018** [cited 2020 Dec 21]; 73:320–330. Available from: /pmc/articles/PMC6191930/?report=abstract

2. Ritchie ME, Phipson B, Wu D, et al. limma powers differential expression analyses for RNA-sequencing and microarray studies. Nucleic Acids Res [Internet]. Oxford University Press; **2015** [cited 2018 Jul 30]; 43(7):e47–e47. Available from: http://academic.oup.com/nar/article/43/7/e47/2414268/limma-powers-differential-expression-analyses-for

3. R Core Team. R: A Language and Environment for Statistical Computing [Internet]. Vienna, Austria: R Foundation for Statistical Computing; 2019. Available from: https://www.r-project.org/

4. Althouse AD. Adjust for Multiple Comparisons? It’s Not That Simple. Ann Thorac Surg [Internet]. Elsevier USA; **2016** [cited 2020 Dec 16]; 101(5):1644–1645. Available from: http://dx.doi.org/10.1016/j.athoracsur.2015.11.024

5. Subramanian A, Tamayo P, Mootha VK, et al. Gene set enrichment analysis: A knowledge-based approach for interpreting genome-wide expression profiles. Proc Natl Acad Sci U S A [Internet]. **2005** [cited 2018 Jun 6]; 102(43):15545–15550. Available from: https://dartmouth.sharepoint.com/sites/networkJC/Lists/Papers/Attachments/3/Gene set enrichment analysis- A knowledge-based approach for interpreting genome-wide expression profiles.pdf

6. Korotkevich G, Sukhov V, Budin N, Shpak B, Artyomov MN, Sergushichev A. Fast gene set enrichment analysis. bioRxiv [Internet]. Cold Spring Harbor Laboratory; **2021** [cited 2023 Mar 14]; :060012. Available from: https://www.biorxiv.org/content/10.1101/060012v3

7. Merico D, Isserlin R, Stueker O, Emili A, Bader GD. Enrichment map: A network-based method for gene-set enrichment visualization and interpretation. Ravasi T, editor. PLoS One [Internet]. Public Library of Science; **2010** [cited 2018 Jul 30]; 5(11):e13984. Available from: http://dx.plos.org/10.1371/journal.pone.0013984

8. Wilcoxon F. Individual Comparisons by Ranking Methods. Biometrics Bull. JSTOR; **1945**; 1(6):80.

9. Newman AM, Steen CB, Liu CL, et al. Determining cell type abundance and expression from bulk tissues with digital cytometry. Nat Biotechnol [Internet]. Nature Research; **2019** [cited 2021 Mar 11]; 37(7):773–782. Available from: https://doi.org/10.1038/s41587-019-0114-2

10. Student. The probable error of a mean. Biometrika. JSTOR; **1908**; :1–25.

11. Langfelder P, Horvath S. WGCNA: An R package for weighted correlation network analysis. BMC Bioinformatics [Internet]. BioMed Central; **2008** [cited 2020 Dec 24]; 9(1):559. Available from: https://bmcbioinformatics.biomedcentral.com/articles/10.1186/1471-2105-9-559

12. Raudvere U, Kolberg L, Kuzmin I, et al. G:Profiler: A web server for functional enrichment analysis and conversions of gene lists (2019 update). Nucleic Acids Res [Internet]. Oxford University Press; **2019** [cited 2020 Dec 30]; 47(W1):W191–W198. Available from: https://academic.oup.com/nar/article/47/W1/W191/5486750

13. Kanehisa M, Furumichi M, Tanabe M, Sato Y, Morishima K. KEGG: New perspectives on genomes, pathways, diseases and drugs. Nucleic Acids Res [Internet]. Oxford University Press; **2017** [cited 2018 Oct 3]; 45(D1):D353–D361. Available from: https://academic.oup.com/nar/article-lookup/doi/10.1093/nar/gkw1092

14. Kanehisa M, Goto S. KEGG: Kyoto Encyclopedia of Genes and Genomes [Internet]. Nucleic Acids Res. Oxford University Press; 2000 [cited 2021 Jan 2]. p. 27–30. Available from: https://pubmed.ncbi.nlm.nih.gov/10592173/

15. Wu G, Haw R. Functional interaction network construction and analysis for disease discovery. Methods Mol Biol [Internet]. Humana Press Inc.; 2017 [cited 2021 Jan 2]. p. 235–253. Available from: https://pubmed.ncbi.nlm.nih.gov/28150241/

16. Fabregat A, Sidiropoulos K, Viteri G, et al. Reactome pathway analysis: A high-performance in-memory approach. BMC Bioinformatics [Internet]. BioMed Central Ltd.; **2017** [cited 2021 Jan 2]; 18(1). Available from: https://pubmed.ncbi.nlm.nih.gov/28249561/

17. Ashburner M, Ball CA, Blake JA, et al. Gene Ontology: tool for the unification of biology. Nat Genet [Internet]. **2000** [cited 2018 May 24]; 25(1):25–29. Available from: http://www.ncbi.nlm.nih.gov/pubmed/10802651

18. Kelder T, Iersel MP Van, Hanspers K, et al. WikiPathways: Building research communities on biological pathways. Nucleic Acids Res [Internet]. Oxford Academic; **2012** [cited 2021 Jan 2]; 40(D1):D1301–D1307. Available from: http://www.wikipathways.org

19. Kutmon M, Riutta A, Nunes N, et al. WikiPathways: Capturing the full diversity of pathway knowledge. Nucleic Acids Res [Internet]. Oxford University Press; **2016** [cited 2021 Jan 2]; 44(D1):D488–D494. Available from: https://academic.oup.com/nar/article/44/D1/D488/2502580

20. Slenter DN, Kutmon M, Hanspers K, et al. WikiPathways: A multifaceted pathway database bridging metabolomics to other omics research. Nucleic Acids Res [Internet]. Oxford University Press; **2018** [cited 2021 Jan 2]; 46(D1):D661–D667. Available from: https://academic.oup.com/nar/article/46/D1/D661/4612963

21. Matys V, Kel-Margoulis O V., Fricke E, et al. TRANSFAC and its module TRANSCompel: transcriptional gene regulation in eukaryotes. Nucleic Acids Res [Internet]. Nucleic Acids Res; **2006** [cited 2021 Jan 2]; 34(Database issue). Available from: https://pubmed.ncbi.nlm.nih.gov/16381825/

22. Hsu S Da, Lin FM, Wu WY, et al. MiRTarBase: A database curates experimentally validated microRNA-target interactions. Nucleic Acids Res [Internet]. Oxford University Press; **2011** [cited 2021 Jan 2]; 39(SUPPL. 1):D163. Available from: /pmc/articles/PMC3013699/?report=abstract

23. Huang HY, Lin YCD, Li J, et al. MiRTarBase 2020: Updates to the experimentally validated microRNA-target interaction database. Nucleic Acids Res [Internet]. Oxford University Press; **2020** [cited 2021 Jan 2]; 48(D1):D148–D154. Available from: https://academic.oup.com/nar/article/48/D1/D148/5606625

24. Thul PJ, Akesson L, Wiking M, et al. A subcellular map of the human proteome. Science (80- ) [Internet]. American Association for the Advancement of Science; **2017** [cited 2021 Jan 2]; 356(6340). Available from: https://science.sciencemag.org/content/356/6340/eaal3321

25. Uhlen M, Fagerberg L, Hallstrom BM, et al. Tissue-based map of the human proteome. Science (80- ) [Internet]. American Association for the Advancement of Science; **2015** [cited 2021 Jan 2]; 347(6220):1260419–1260419. Available from: https://www.sciencemag.org/lookup/doi/10.1126/science.1260419

26. Uhlen M, Zhang C, Lee S, et al. A pathology atlas of the human cancer transcriptome. Science (80- ) [Internet]. American Association for the Advancement of Science; **2017** [cited 2021 Jan 2]; 357(6352). Available from: http://science.sciencemag.org/

27. Navani S. The human protein atlas [Internet]. J. Obstet. Gynecol. India. 2011 [cited 2021 Jan 2]. p. 27–31. Available from: https://www.proteinatlas.org/

28. Ruepp A, Brauner B, Dunger-Kaltenbach I, et al. CORUM: The comprehensive resource of mammalian protein complexes. Nucleic Acids Res [Internet]. Oxford University Press; **2008** [cited 2021 Jan 2]; 36(SUPPL. 1):D646. Available from: /pmc/articles/PMC2238909/?report=abstract

29. Köhler S, Carmody L, Vasilevsky N, et al. Expansion of the Human Phenotype Ontology (HPO) knowledge base and resources. Nucleic Acids Res [Internet]. Oxford University Press; **2019** [cited 2021 Jan 2]; 47(D1):D1018–D1027. Available from: https://academic.oup.com/nar/article/47/D1/D1018/5198478

30. Harkness WL. Properties of the Extended Hypergeometric Distribution. Ann Math Stat [Internet]. Institute of Mathematical Statistics; **1965** [cited 2021 Jan 2]; 36(3):938–945. Available from: https://projecteuclid.org/euclid.aoms/1177700066

31. Shannon P. Cytoscape: A Software Environment for Integrated Models of Biomolecular Interaction Networks. Genome Res [Internet]. **2003** [cited 2018 Jul 30]; 13(11):2498–2504. Available from: http://www.genome.org/cgi/doi/10.1101/gr.1239303

32. Anderson ST, Kaforou M, Brent AJ, et al. Diagnosis of Childhood Tuberculosis and Host RNA Expression in Africa. N Engl J Med [Internet]. **2014** [cited 2018 Jul 30]; 370(18):1712–1723. Available from: http://www.ncbi.nlm.nih.gov/pubmed/24785206

33. Edgar R, Domrachev M, Lash AE. Gene Expression Omnibus: NCBI gene expression and hybridization array data repository. Nucleic Acids Res [Internet]. Oxford University Press; **2002** [cited 2018 May 26]; 30(1):207–210. Available from: https://academic.oup.com/nar/article-lookup/doi/10.1093/nar/30.1.207

34. Benjamini Y, Hochberg Y. Controlling the False Discovery Rate: A Practical and Powerful Approach to Multiple Testing. J R Stat Soc Ser B [Internet]. Wiley; **1995** [cited 2020 Nov 13]; 57(1):289–300. Available from: https://rss.onlinelibrary.wiley.com/doi/full/10.1111/j.2517-6161.1995.tb02031.x

35. Pearson K. LIII. On lines and planes of closest fit to systems of points in space . London, Edinburgh, Dublin Philos Mag J Sci [Internet]. Informa UK Limited; **1901** [cited 2021 Jan 4]; 2(11):559–572. Available from: https://www.tandfonline.com/doi/abs/10.1080/14786440109462720
