# Supplementary material for: Gene Expression in Cord Blood and Tuberculosis in Early Childhood: A Nested Case-Control Study in a South African Birth Cohort
Source: Clin Infect Dis. 2023 May 5;77(3):438–49. doi: 10.1093/cid/ciad268 (PMC10425199; doi:10.1093/cid/ciad268)
Supplement: ciad268_Supplementary_Data [file ciad268_supplementary_data.zip › SupplementaryFigures.docx]

Gene expression in cord blood and tuberculosis in early childhood:

A nested case-control study in a South African birth cohort

Supplementary Figures

Carly A. Bobak, Maresa Botha, Lesley Workman, Jane E. Hill, Mark Nicol,^,^John W. Holloway, Dan J Stein, Leonardo Martinez, Heather J Zar

Supplementary Figure 1: A volcano plot depicting differential gene results between infants who were diagnosed with TB before 5 years of age and those who were not. Upregulated genes are shown in red and down regulated genes are shown in blue.


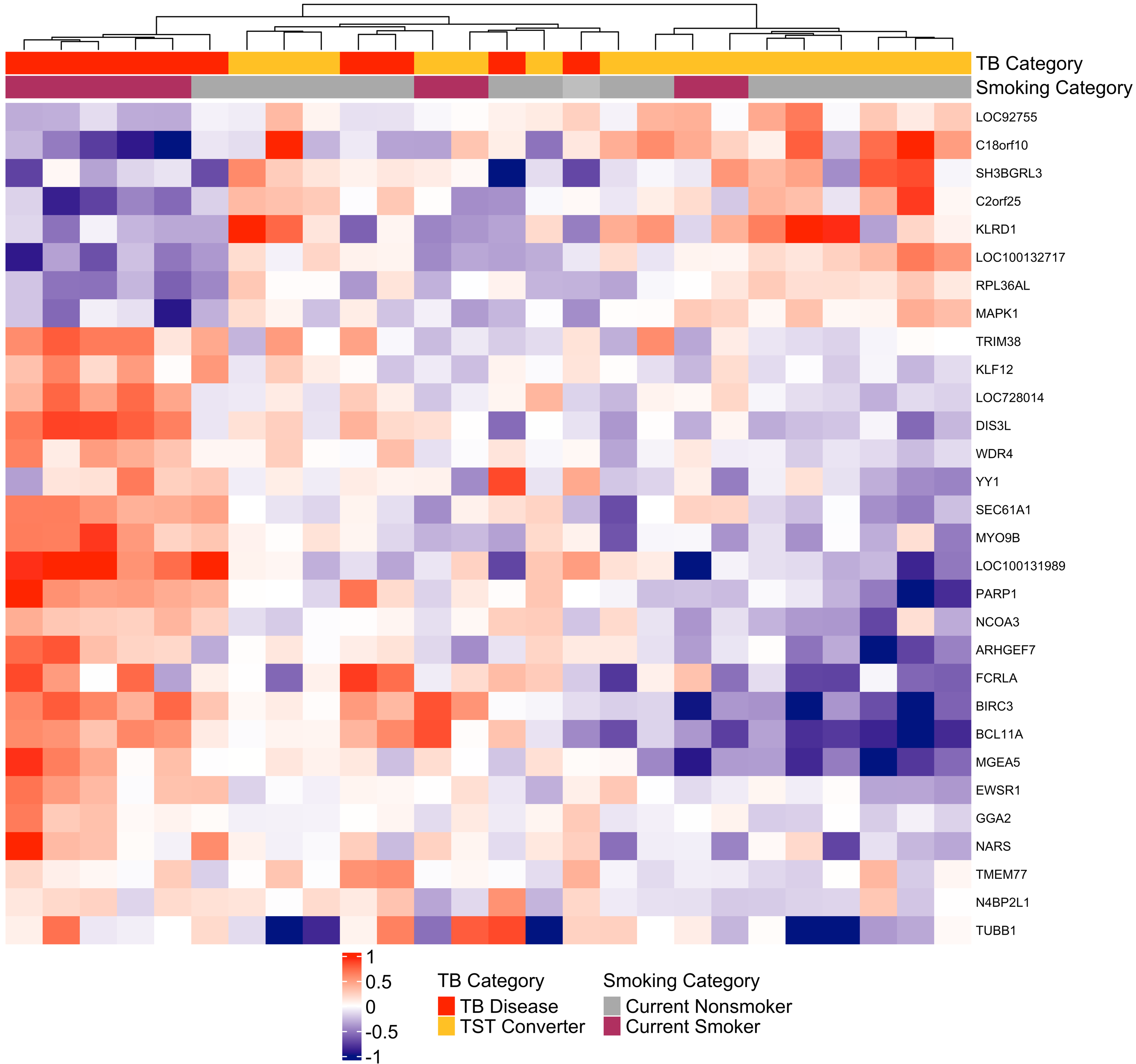


Supplementary Figure 2: Heatmap displaying differentially expressed genes between children who were early TST converters who did or did not develop tuberculosis prior to five years of age. The annotation bar shows both TB category (red TB, yellow for TST conversion) and smoking category (mother was a current smoker at time of enrollment in maroon, current non-smoker in grey, 1 missing value in lighter grey). Expression values are median centered. Both columns and rows were clustered using Canberra distance.

Supplementary Figure 3: An enrichment map of overlapping pathways either enriched or depleted in the measured TB outcomes or maternal current smoking status. Nodes represent known gene sets, and edges between nodes indicate a significant overlap of genes between gene sets (similarity score >= 0.63). Nodes are divided into 4 sections aligning with the 4 measured outcomes, and color is indicative of either depletion (blue) or enrichment (red).

C

B

A

Supplementary Figure 4: The module significance across all 3 TB outcomes. Module significance is measured by comparing the mean -log10 pvalue of each gene in each association across modules. (A) The early converision signature across biologically relevant modules, (B) The early development of TB disease across modules, and (C) the early development of TB disease within infants with early TST conversion.

Supplementary Figure 5: The full regression results across all DGE associations and modules. The first column is the DGE associated with early TST conversion, the second is the DGE associated with TB diagnosis before the age of 5, and the third is TB diagnosis before the age of 5 within early TST converters. The y-axis in each plot is the correlation between the gene expression value and the outcome of interest, while the x-axis is the modular gene connectivity (kME).


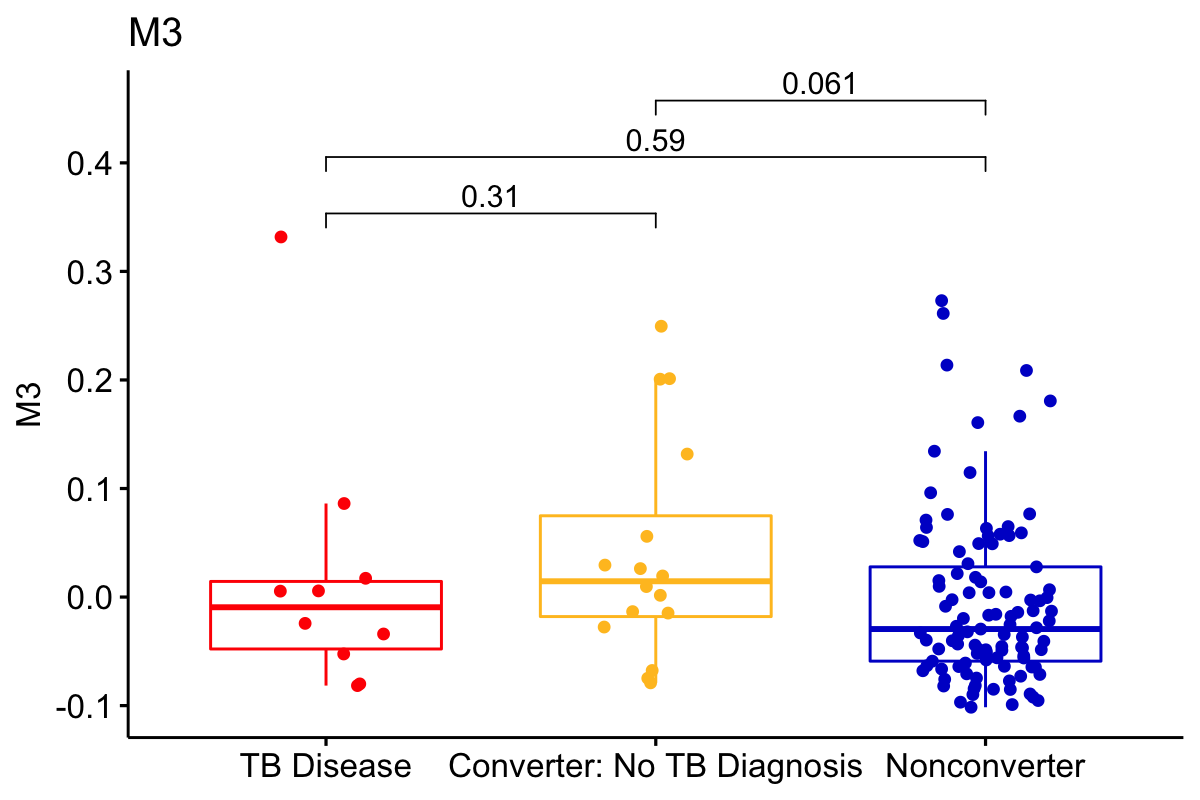

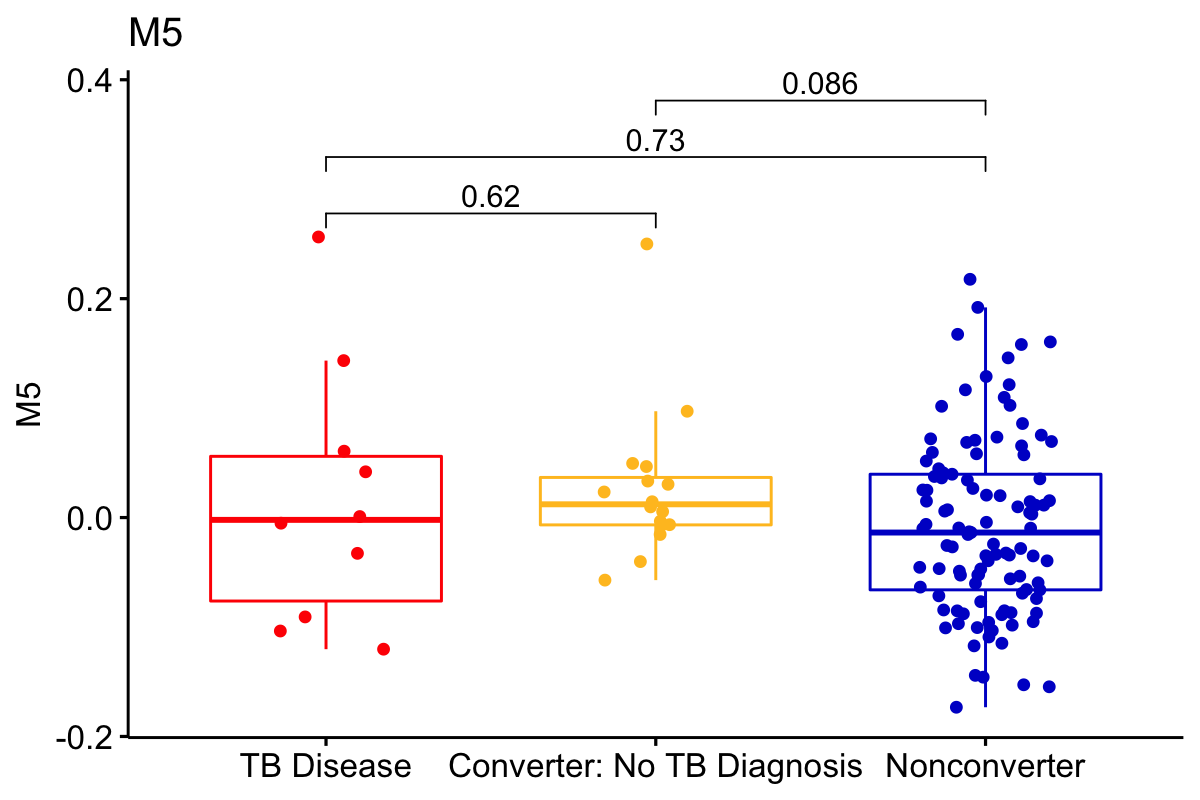

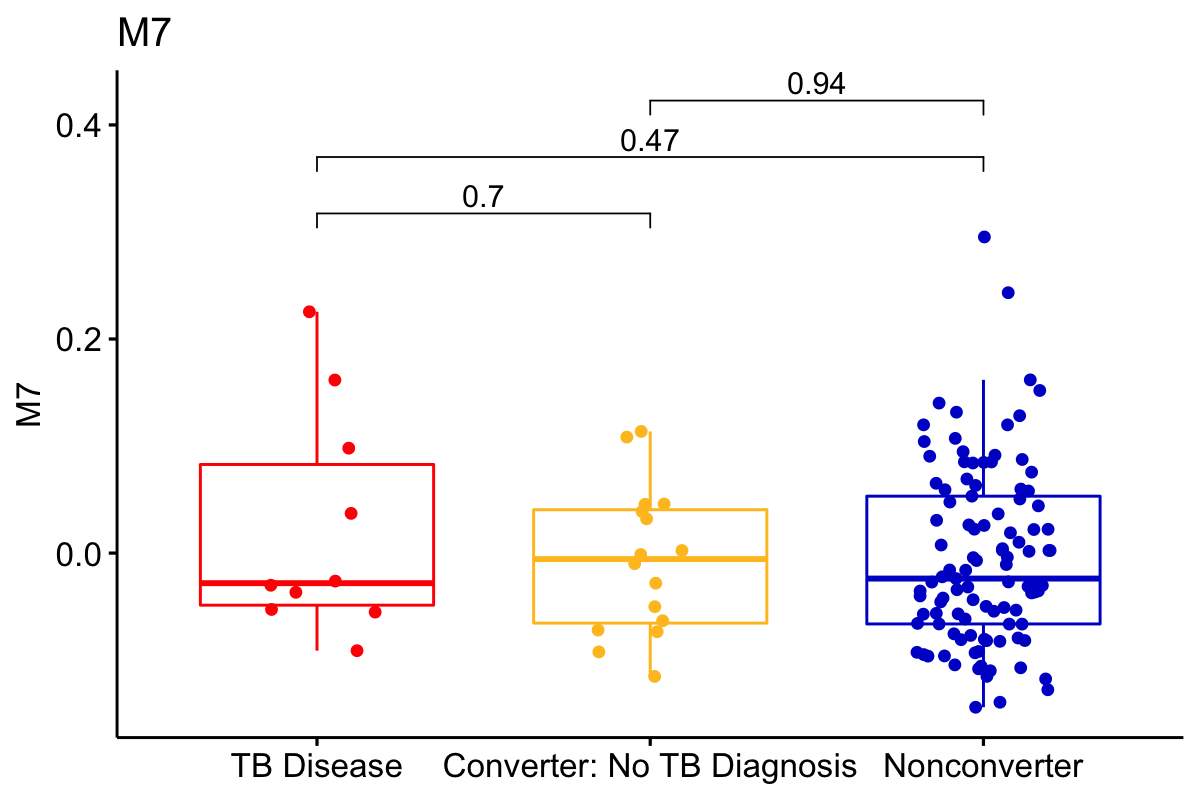

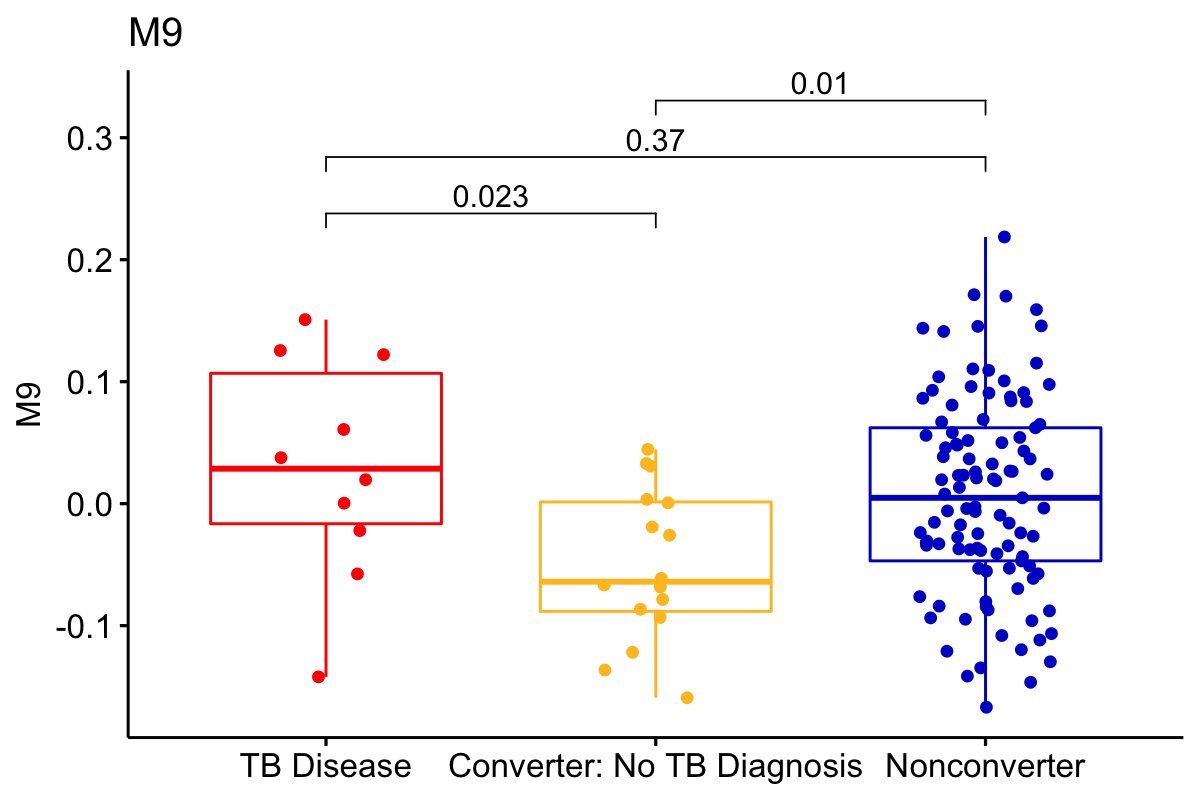

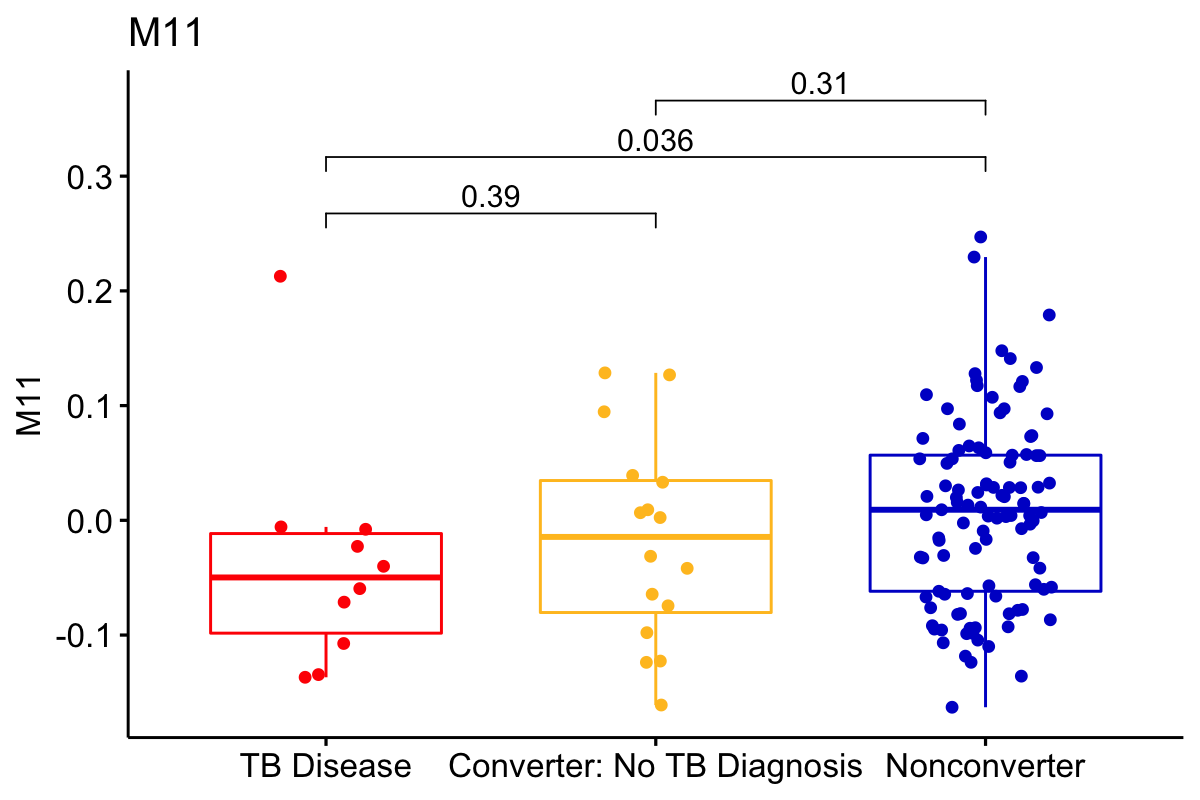

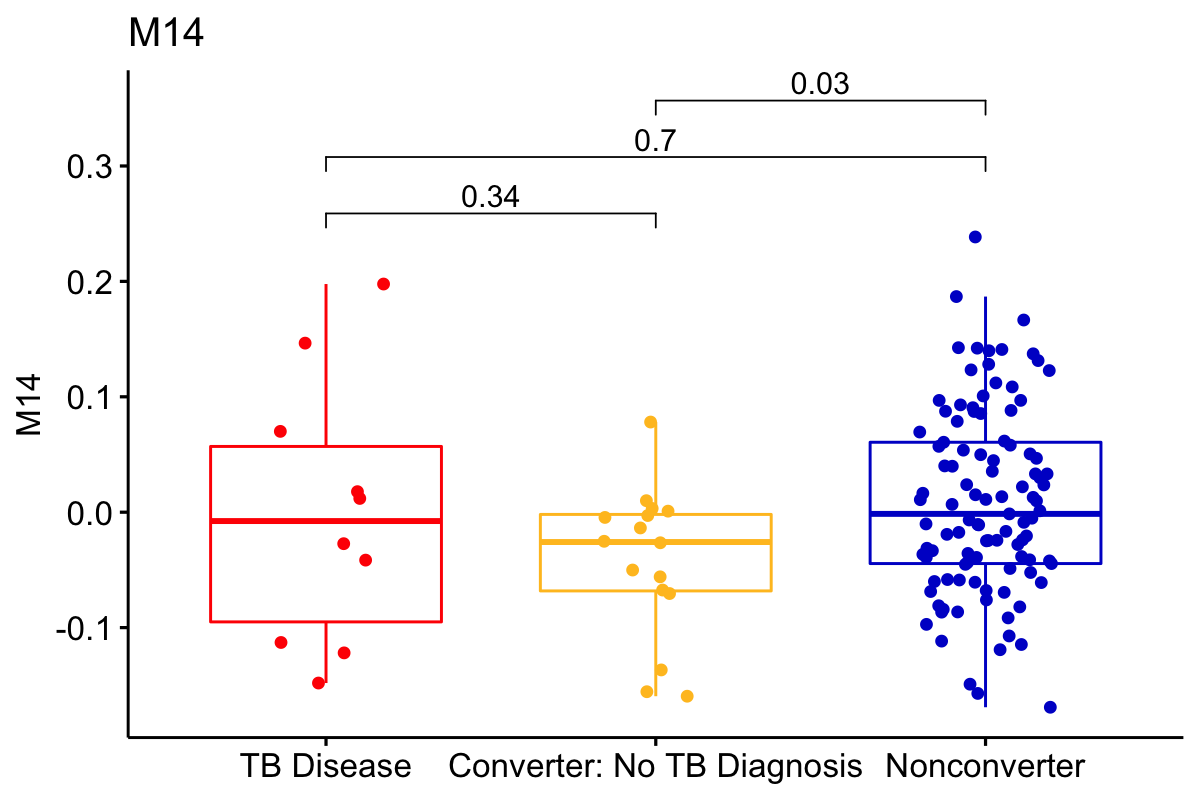


Supplementary Figure 6: The module eigengene across TB outcomes of interest, where TB Disease represents infants who develop TB disease in the first 5 years of age among those who are early TST converters, Converter:No TB Diagnosis represents early converter who did not have a positive TB diagnosis before 5 years of age, and non-converters are those infants who have neither a TB diagnosis before 5 years of age nor positive TST test within the first 36 months of life. The p-values shown are from a Wilcoxon rank sum test.

Supplementary Figure 7: The full enrichment map for all Modules statistically associated with measured TB outcomes. Nodes represent known biological pathways, and edges between the nodes indicate the overlap of genes between pathways. M5 is shown in salmon, M9 in brown, M11 in royal blue and M14 in purple.

Supplemental Figure 8: Validation results from Anderson cohorts. (A) and (B) show the overlap of genes differentially expressed in both the Malawai and Kenyan cohorts; where (A) is our genes associated with TST convergence and (B) our genes associated with TB disease. (C) and (D) show the correlation between the M11 gene connectivity measured in the cord blood and the significance of genes in Anderson between samples with LTBI and TB. (E) and (F) show the M11 eigengene, recalculated in Anderson across measured genes, and its correlation with possible disease outcomes.
